# Supplementary material for: Genomic and Environmental Factors Shape the Active Gill Bacterial Community of an Amazonian Teleost Holobiont
Source: Microbiol Spectr. 2022 Nov 29;10(6):e02064-22. doi: 10.1128/spectrum.02064-22 (PMC9769777; doi:10.1128/spectrum.02064-22)
Supplement: Supplemental file 1 — Supplemental text, Fig. S1 to S5, and Tables S1 to S6. Download spectrum.02064-22-s0001.pdf, PDF file, 0.8 MB [file spectrum.02064-22-s0001.pdf]

Supplementary material

Genomic and environmental factors shape the active gill bacterial community of an Amazonian teleost holobiont

Sylvain, François-Étienne; Leroux, Nicolas; Normandeau, Éric; Holland, Aleicia; Bouslama, Sidki; Mercier, Pierre-Luc; Val, Adalberto Luis; Derome, Nicolas

Supplementary methods

Details on 16S rRNA library preparation

The first PCR was performed with primers specific to the V3-V4 region of the 16S rRNA gene (primers 347F and 803R; Nossa *et al*., 2010), which were tailed on the 5' end with part of the Illumina TruSeq adaptors (Oligonucleotide sequences © 2007-2013 Illumina, Inc. All rights reserved).

The following oligonucleotide sequences were used for amplification for the first PCR (actual primer sequence is in bold, the rest corresponds to the adapter sequence):

Forward primer (347F):
5'-ACACTCTTTCCCTACACGACGCTCTTCCGATCT**GGAGGCAGCAGTRRGGAAT**-3',
Reverse primer (803R):
5'-GTGACTGGAGTTCAGACGTGTGCTCTTCCGATCT**CTACCRGGGTATCTAATCC**-3',

Then, a second PCR was performed to attach remaining adaptor sequence (the regions that anneal to the flowcell and library specific barcodes).

Generic forward second-PCR primer:
5'-AATGATACGGCGACCACCGAGATCTACAC[index1]ACACTCTTTCCCTACACGAC-3'
Generic reverse second-PCR primer:
5'-CAAGCAGAAGACGGCATACGAGAT[index2]GTGACTGGAGTTCAGACGTGT-3'.

Supplementary figures

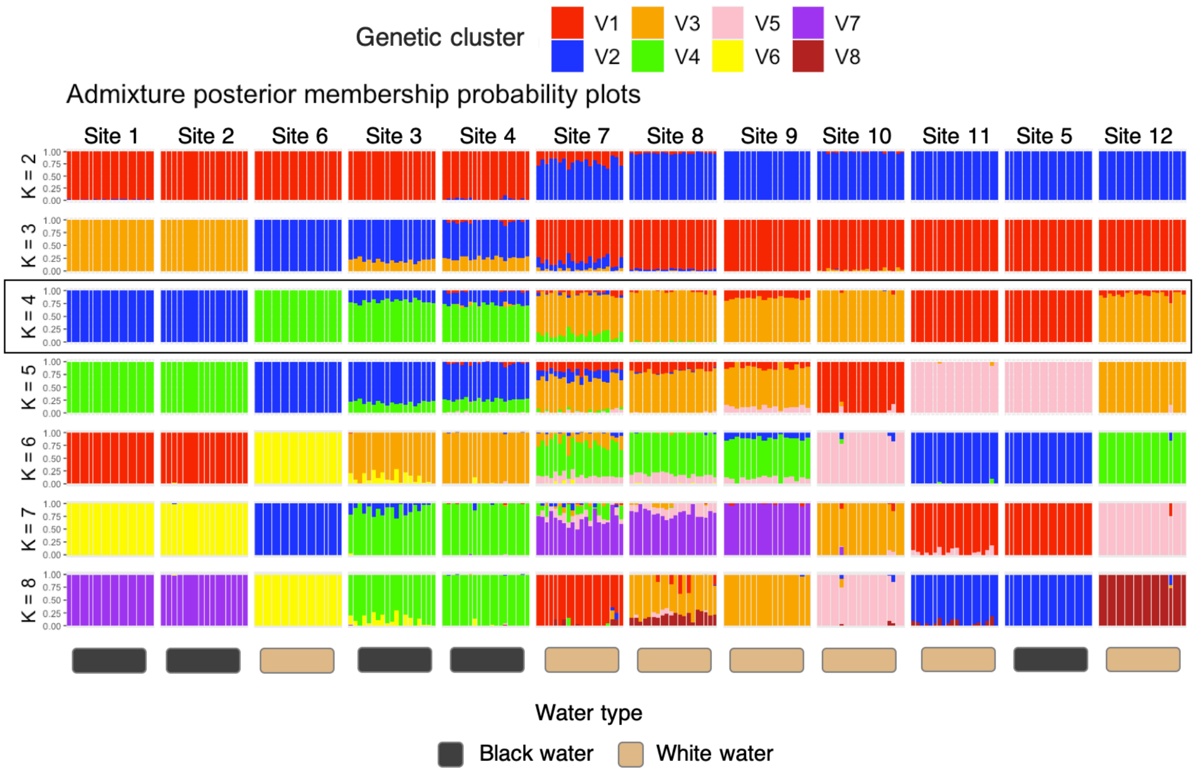

Suppl. Figure 1: Modified from Suppl. Fig. 1 in Leroux *et al*. 2022. Posterior membership probability plots from Admixture based on the 41,268 SNPs and considering two to eight genetic clusters (K). Each individual (n = 231) is represented by a single vertical line colored proportionally to its posterior membership probability to one of the eight genetic clusters considered for this analysis. Samples are wrapped by sampling sites. The cross-validation (CV) error values from Admixture are very close for K = 3 (CV = 0.20966) and K = 4 (CV = 0.20997). The optimal number of genetic clusters was determined to be K = 4 because the goodness-of-fit (BIC) values from find.cluster (Adegenet) reduced more slowly at the fourth cluster (Suppl. Fig. 3 in Leroux *et al*. 2022). Furthermore, the posterior membership probability plots stopped forming biologically significant clusters after K = 4, differentiating only one sampling site at the time when adding more clusters (Suppl. Fig. 1 in Leroux *et al*. 2022).

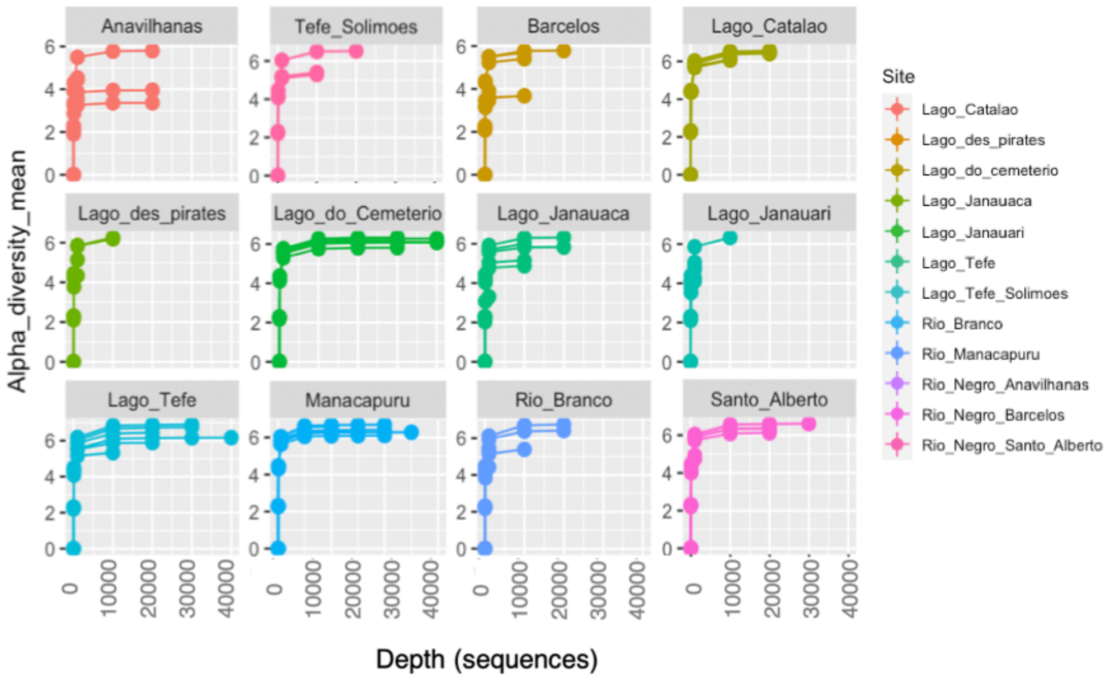

Suppl. Figure 2: Rarefaction plots of the samples for each sampling site, for the **bacterioplankton communities**. The rarefaction analysis was based on the Shannon diversity for each sample group, according to the sequencing depth (number of sequences used)

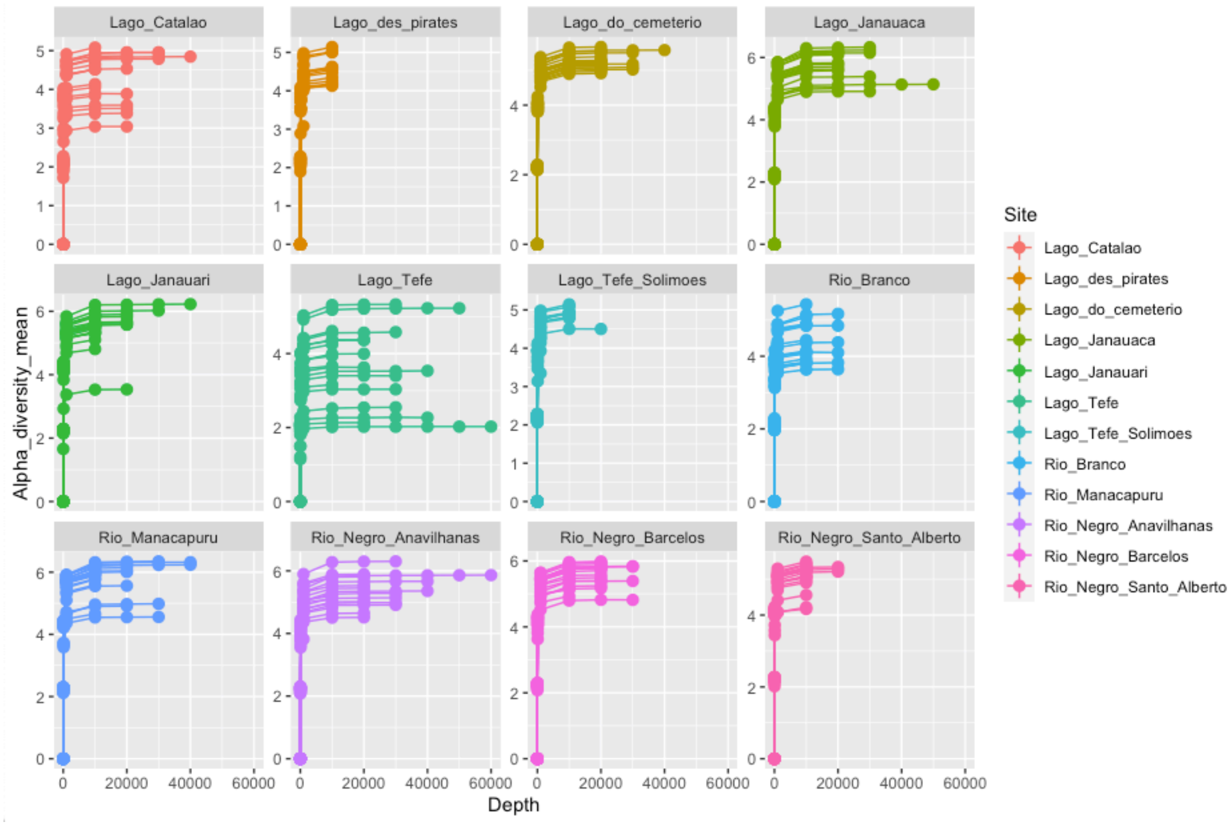

Suppl. Figure 3: Rarefaction plots of the samples for each sampling site, for the **gill microbiomes**. The rarefaction analysis was based on the Shannon diversity for each sample group, according to the sequencing depth (number of sequences used)

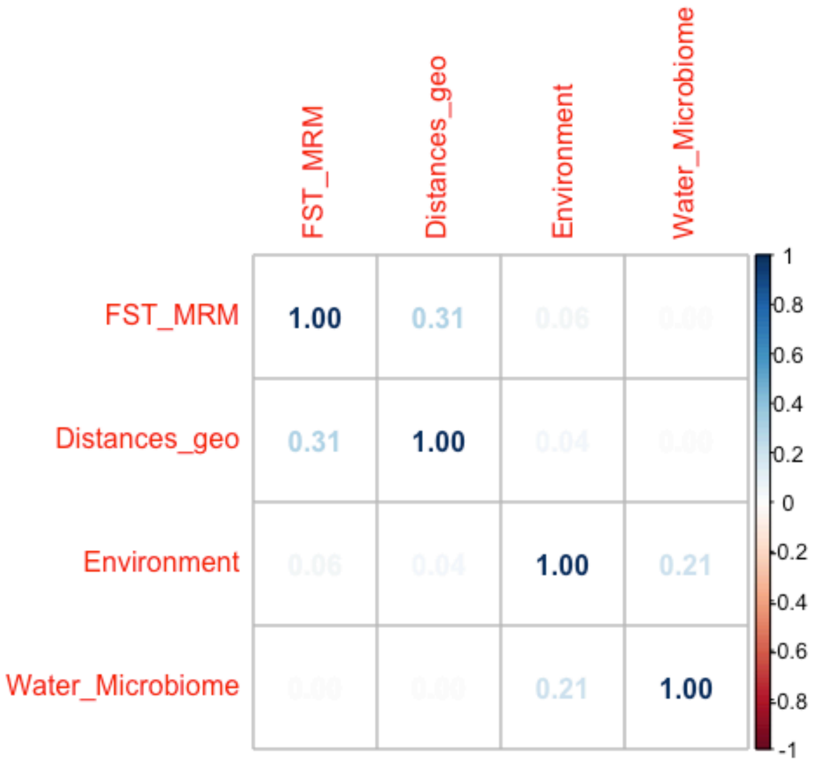

Suppl. Figure 4: Analysis of the covariation (Spearman correlations) between the factors considered in the linear mixed-effect models shown in Fig. 6. “FST_MRM” stands for the FST values calculated from the genetic background of hosts from different sampling sites, “Distances_geo” stands for the normalized Euclidean distances of the geographical distances between sampling sites, “Environment” stands for the normalized Euclidean distances between sampling sites calculated from the set of 34 environmental parameters measured in this study, and “Water_Microbiome” stands for the Bray-Curtis dissimilarity between bacterioplankton samples from different sampling sites.

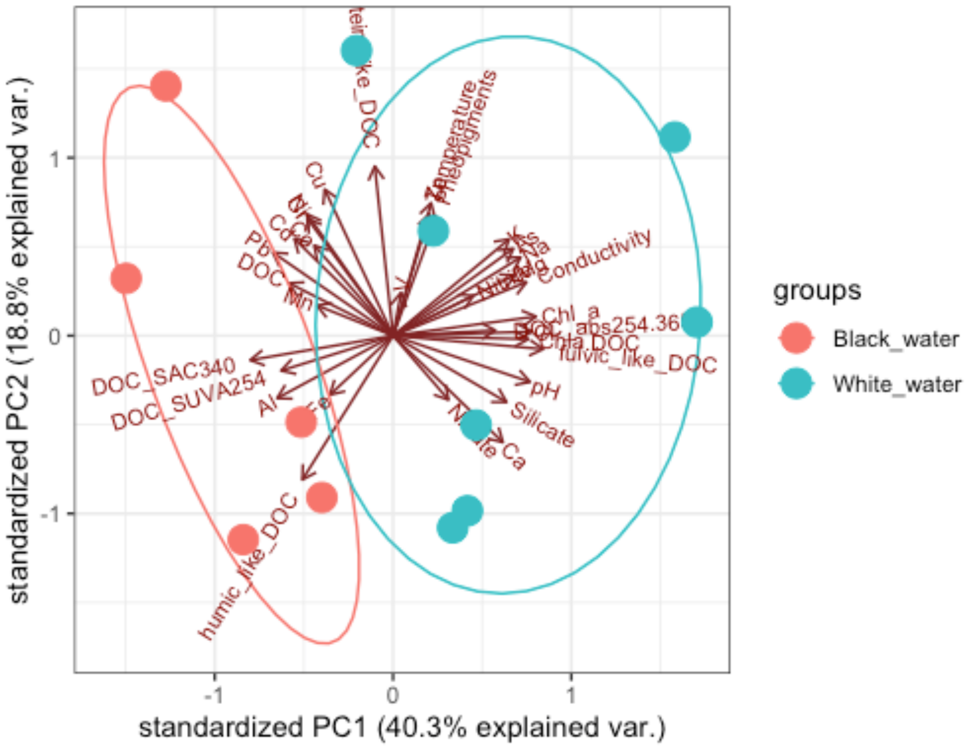

Suppl. Figure 5: PCA of the 34 environmental parameters measured in this study. Each point corresponds to a sampling site. The points are colored according to the water color found at the site. No selection of environmental variables was done prior to plotting – this PCA displays all the measured environmental variables, including those that potentially significantly co-vary.

Supplementary tables

Suppl. Table 1: Site identification, water color, geographical coordinates, ecosystem type, sampling time for each sampling site.

Site characteristics							
Site #	Site name	Water color	Genetic cluster	GPS S	GPS W	Ecosystem	Sampling time
1	Rio Negro - Barcelos	Black	GC1	0°50'50.8"S	62°57'40.3"W	River	11/2018
2	Rio Negro - Santo Alberto	Black	GC1	1°23'29.8"S	61°59'35.3"W	River	10/2019
3	Rio Negro - Anavilhanas	Black	GC2	2°41'46.1"S	60°46'33.3"W	River	10/2018
4	Lago do cemeterio	Black	GC2	3°02'16.6"S	60°32'42.7"W	Lake	10/2019
5	Lago Téfé	Black	GC4	3°27'55.2"S	64°53'13.2"W	Lake	11/2019
6	Rio Branco	White	GC2	1°19'05.7"S	61°52'34.7"W	River	10/2019
7	Lago Janauari	White	GC3	3°12'03.4"S	60°03'10.1"W	Lake	10/2018
8	Lago Catalão	White	GC3	3°09'56.4"S	59°54'38.4"W	Lake	10/2018
9	Lago Janauaca	White	GC3	3°23'37.5"S	60°19'52.6"W	Lake	11/2018
10	Rio Manacapuru	White	GC3	3°16'16.9"S	60°42'03.2"W	River	11/2018
11	Lago Téfé-Solimões	White	GC4	3°21'07.4"S	64°40'21.4"W	Lake	11/2019
12	Lago des pirates	White	GC3	3°15'19.2"S	64°41'44.3"W	Lake	11/2019

Suppl. Table 2: Dissolved organic carbon (DOC) quantity and quality characterization[1].

Site #	DOC characteristics						
	DOC conc.	SAC340	SUVA254	Abs 254/365	% humic DOC	% fulvic DOC	% protein DOC
1	10.9	39.5	4.5	3.8	56.7	29.5	13.8
2	11.7	33.5	3.7	3.6	60.3	32.6	7.1
3	11.4	30.5	3.6	3.8	47.2	30.3	22.5
4	9.8	18.9	2.4	3.8	52.3	41.0	6.7
5	7.1	29.1	3.4	4.0	54.2	37.3	8.5
6	6.0	19.1	2.2	4.3	50.8	39.7	9.5
7	7.1	19.1	1.4	2.2	34.7	36.2	29.1
8	9.1	11.7	2.1	6.4	37.5	45.6	16.8
9	5.7	20.0	2.6	4.2	50.6	40.4	9.0
10	8.0	22.1	3.0	4.6	46.2	41.8	12.0
11	5.7	20.1	2.6	3.8	49.0	39.3	11.7
12	6.5	14.2	2.2	4.7	43.9	45.3	10.8

***1:** "DOC conc." means DOC concentration in mg L^{-1}. SAC340 and SUVA254 are the specific absorbance coefficients indexes of relative DOC aromaticity (higher values indicate that the DOC is more aromatic). Abs254/365 is the index of molecular weight: lower values indicate higher DOC molecular weights.

Suppl. Table 3: Concentrations of free ions and nutrients.

Site #	Water color	Ions: mg L^{-1}					Nutrients: umol L^{-1}		
		Na^{+}	Mg^{+2}	K^{+}	Ca^{+2}	Cl^{-}	Nitrite	Nitrate	Silicate
1	Black	0.46	0.12	0.42	0.04	0.11	0.11	3.20	64.41
2	Black	0.25	0.09	0.33	0.49	1.16	0.10	2.87	92.32
3	Black	1.80	0.26	0.65	0.08	0.32	0.09	4.36	72.55
4	Black	0.23	0.05	0.14	0.37	0.64	0.01	0.47	76.82
5	Black	0.87	0.19	0.56	0.82	0.53	0.08	4.09	217.19
6	White	1.15	0.43	0.70	0.93	1.10	0.04	8.23	180.48
7	White	1.99	0.20	0.79	0.06	1.47	0.19	1.31	98.31
8	White	4.56	3.76	1.71	0.83	1.75	0.09	0.56	242.31
9	White	3.32	1.00	1.07	0.44	2.17	0.13	20.45	156.51
10	White	4.91	0.14	1.45	0.05	1.43	0.12	1.53	126.01
11	White	1.95	0.21	0.28	1.11	1.29	0.03	6.47	326.53
12	White	5.35	1.76	1.28	1.17	3.26	0.61	11.96	222.31

Suppl. Table 4: Primary productivity characterization and measure of several physicochemical parameters.[1]

Site #	Water color	Primary productivity: ug L^{-1}			Physicochemical parameters			
		Chl a	Phaeopig.	Chla/DOC	Temp. (°C)	Cond. (uS)	pH	O_2 (%)
1	Black	0.35	2.43	0.03	31.60	13.10	3.71	92
2	Black	0.73	0.33	0.06	30.60	10.60	4.16	58
3	Black	0.05	0.38	0.00	30.70	13.20	4.24	53
4	Black	1.35	1.44	0.14	32.40	7.20	3.83	76
5	Black	1.82	1.73	0.26	30.00	10.60	4.98	61
6	White	6.21	2.89	1.03	31.00	22.00	6.25	88
7	White	4.62	17.31	0.65	32.90	22.40	4.38	60
8	White	7.14	6.60	0.79	32.90	174.80	5.70	44
9	White	1.35	1.88	0.24	29.30	88.00	6.75	82
10	White	2.78	10.54	0.35	32.60	24.30	5.31	72
11	White	4.41	3.20	0.77	30.30	19.70	6.05	68
12	White	9.05	4.69	1.40	31.90	127.60	7.15	31

***1:** “Chl a” means the concentration of chlorophyll a; “Phaeopig.” means the concentration of phaeopigments; “Chla/DOC” is a ratio of the concentration of chlorophyll a divided by the concentration of DOC; “Temp. °C” means the temperature in ° Celsius; “Cond. uS” means the conductivity in microsiemens; “% O_2” means the percentage of saturation of dissolved oxygen.

Suppl. Table 5: Concentration of dissolved metals in ug/L.

Site #	Water color	Metals (ug/l)											
		Al	V	Cr	Mn	Fe	Co	Ni	Cu	Zn	As	Cd	Pb
1	Black	137.75	0.38	0.30	7.38	166.63	0.13	1.93	10.36	33.48	0.16	0.09	1.43
2	Black	150.00	0.10	0.05	5.90	160.00	0.10	0.15	0.30	11.00	0.05	0.02	0.27
3	Black	36.33	0.34	0.37	9.24	142.38	0.28	3.23	9.25	72.92	0.48	0.21	1.11
4	Black	87.00	0.30	0.05	4.60	100.00	0.10	0.33	1.90	9.00	0.08	0.13	0.30
5	Black	62.00	0.10	0.33	13.00	220.00	0.10	0.52	0.60	4.40	0.19	0.02	0.12
6	White	38.00	0.20	0.05	0.51	230.00	0.10	0.14	0.80	2.60	0.07	0.02	0.26
7	White	65.50	0.78	0.40	9.85	269.28	0.10	0.85	16.19	44.15	0.47	0.06	0.67
8	White	1.81	0.17	0.10	0.61	5.84	0.10	0.48	2.20	171.78	0.99	0.02	0.03
9	White	28.02	1.45	0.09	11.25	166.97	0.10	0.58	2.73	1.83	0.70	0.03	0.25
10	White	13.47	0.85	0.21	4.64	97.85	0.10	1.12	2.11	25.85	0.38	0.08	0.16
11	White	49.00	0.30	0.11	0.68	250.00	0.10	0.41	0.50	2.70	0.27	0.02	0.21
12	White	27.00	0.20	0.06	4.60	82.00	0.10	0.60	1.70	8.10	1.30	0.02	0.11

Suppl. Table 6: Envfit results.

Parameters	Bacterioplankton			Gill microbiome		
	r2	Pr(>r)	Significance	r2	Pr(>r)	Significance
DOC	0.0453	0.227		0.0908	0.001	***
DOC_SAC340	0.0227	0.493		0.1731	0.001	***
DOC_SUVA254	0.1696	0.002	**	0.1235	0.001	***
DOC_abs254.365	0.1967	0.006	**	0.2553	0.001	***
humic_like_DOC	0.189	0.001	***	0.1288	0.001	***
fulvic_like_DOC	0.0033	0.924		0.1425	0.001	***
protein_like_DOC	0.2717	0.001	***	0.0307	0.023	*
Na	0.0014	0.955		0.0823	0.001	***
Mg	0.0151	0.439		0.2627	0.001	***
K	0.0005	0.989		0.1607	0.001	***
Ca	0.0464	0.216		0.1361	0.001	***
Cl	0.0056	0.826		0.0301	0.035	*
Nitrite	0.0081	0.538		0.0172	0.127	
Nitrate	0.0178	0.47		0.0086	0.359	
Silicate	0.0139	0.718		0.1129	0.001	***
Chl_a	0.027	0.445		0.231	0.001	***
Pheopigments	0.3593	0.001	***	0.084	0.001	***
Chla.DOC	0.0295	0.372		0.1729	0.001	***
Temperature	0.0726	0.066	.	0.0368	0.01	**
Conductivity	0.0131	0.672		0.1662	0.001	***
pH	0.0113	0.746		0.1042	0.001	***
Al	0.0001	1		0.1677	0.001	***
V	0.0397	0.203		0.1496	0.001	***
Cr	0.1298	0.002	**	0.1338	0.001	***
Mn	0.033	0.412		0.2841	0.001	***
Fe	0.1683	0.001	***	0.0389	0.009	**
Co	0.0135	0.316		0.0358	0.022	*
Ni	0.0034	0.9		0.0853	0.001	***
Cu	0.265	0.002	**	0.0702	0.001	***
Zn	0.0005	0.981		0.1765	0.001	***
As	0.001	0.969		0.0357	0.012	*
Cd	0.0052	0.883		0.1125	0.001	***
Pb	0.0084	0.801		0.0883	0.001	***

Supplementary results

Environmental characterization

The 12 sites considered in this study showed two contrasting physico-chemical profiles corresponding to two Amazonian water types (Fig. 6b, Suppl. Tables 2, 3, 4, 5, and Suppl. Fig. 5). Environmental parameters that were enriched in black water sites were mostly related to metal concentrations, the five most important being: Al (2.97 X the mean concentration in white water sites), Pb (2.69 X), Cd (2.64 X), Ni (2.06 X) and Mn (1.74 X). In contrast, environmental parameters showing the highest values in white water sites were the Mg^{2+} concentration (7.56 X the concentration in black water sites), the chlorophyll a / DOC ratio (7.56 X), conductivity (6.25 X), and the concentrations of chlorophyll a (5.90 X) and phaeopigments (5.34 X). Additional parameters typically associated with water types, such as pH, silicate concentration, DOC concentration, and the relative abundance of humic and fulvic DOC (in %) also showed significant variations between water types (Fig. 6b). Black water sites contained higher amounts of DOC, that was significantly enriched (p-value = 0.006, t = 3.24, df = 13) in the humic-like fraction of greater aromaticity and molecular weight. In contrast, white water sites contained DOC characterized by a high content of fulvic-like components of low aromaticity and molecular weight.

References

Leroux, N., Sylvain, F-É, Normandeau, E, Holland, A., Val, A.L., Derome, N. 2022. Evolution of an Amazonian fish is driven by allopatric divergence rather than ecological divergence. Frontiers in Ecology and Evolution, 10:875961. doi: 10.3389/fevo.2022.875961

Nossa, C. W., Oberdorf, W. E., Yang, L., Aas, J. A., Paster, B. J., Desantis, T. Z., Brodie, E. L., Malamud, D., Poles, M. A., Pei, Z. 2010. Design of 16S rRNA gene primers for 454 pyrosequencing of the human foregut microbiome. World Journal of Gastroenterology, 16:4135-4144. doi:10.3748/wjg.v16.i33.4135
